# Gonadotropin treatment augments postnatal oogenesis and primordial follicle assembly in adult mouse ovaries?

**DOI:** 10.1186/1757-2215-5-32

**Published:** 2012-11-07

**Authors:** Deepa Bhartiya, Kalpana Sriraman, Pranesh Gunjal, Harshada Modak

**Affiliations:** 1 Stem Cell Biology Department, National Institute for Research in Reproductive Health, Mumbai, 400 012, India

**Keywords:** Ovary, PMSG, Stem cells, VSELs, Primordial follicles, Oct-4

## Abstract

**Background:**

Follicle stimulating hormone (FSH) exerts action on both germline and somatic compartment in both ovary and testis although FSH receptors (FSHR) are localized only on the somatic cells namely granulosa cells of growing follicles and Sertoli cells in the seminiferous tubules. High levels of FSH in females are associated with poor ovarian reserve, ovarian hyper stimulation syndrome etc. and at the same time FSH acts as a survival factor during *in vitro* organotypic culture of ovarian cortical strips. Thus a further understanding of FSH action on the ovary is essential. We have earlier reported presence of pluripotent very small embryonic-like stem cells (VSELs express Oct-4A in addition to other pluripotent markers) and their immediate descendants ‘progenitors’ ovarian germ stem cells (OGSCs express Oct-4B in addition to other germ cell markers) in ovarian surface epithelium (OSE) in various mammalian species including mice, rabbit, monkey, sheep and human. Present study was undertaken to investigate the effect of pregnant mare serum gonadotropin (PMSG) on adult mice ovaries with a focus on VSELs, OGSCs, postnatal oogenesis and primordial follicle assembly.

**Methods:**

Ovaries were collected from adult mice during different stages of estrus cycle and after 2 and 7 days of PMSG (5 IU) treatment to study histo-architecture and expression for FSHR, pluripotent stem cells , meiosis and germ cell specific markers.

**Results:**

PMSG treatment resulted in increased FSHR and proliferation as indicated by increased FSHR and PCNA immunostaining in OSE and oocytes of primordial follicles (PF) besides the granulosa cells of large antral follicles. Small 1–2 regions of multilayered OSE invariably associated with a cohort of PF during estrus stage in control ovary were increased to 5–8 regions after PMSG treatment. This was associated with an increase in pluripotent transcripts (Oct-4A, Nanog), meiosis (Scp-3) and germ cells (Oct-4B, Mvh) specific markers. MVH showed positive immuno staining on germ cell nest-like clusters and at places primordial follicles appeared connected through oocytes.

**Conclusions:**

The results of the present study show that gonadotropin (PMSG) treatment to adult mouse leads to increased pluripotent stem cell activity in the ovaries, associated with increased meiosis, appearance of several cohorts of PF and their assembly in close proximity of OSE. This was found associated with the presence of germ cell nests and cytoplasmic continuity of oocytes in PF. We have earlier reported that pluripotent ovarian stem cells in the adult mammalian ovary are the VSELs which give rise to slightly differentiated OGSCs. Thus we propose that gonadotropin through its action on pluripotent VSELs augments neo-oogenesis and PF assembly in adult mouse ovaries.

## Background

Follicle-stimulating hormone (FSH) is a pleiotropic hormone produced by the pituitary and exerts its action on the ovary by inducing proliferation, differentiation and steroidogenesis in the granulosa cells of growing pre-ovulatory follicles. The initial development of primordial follicles (PF) is believed to be FSH independent [1]. However, several reports have shown that appreciable amount of FSH is present in serum of perinatal mice [2], rats [3], hamsters [4,5] and humans [6]. Elevated FSH levels are found to be associated with altered ovarian function including diminished ovarian reserve, pre-mature ovarian failure, infertility, ovarian hyper-stimulation syndrome, poor quality of eggs, menopause [7,8] and also a huge body of literature exists suggesting an association with ovarian cancers [9,10]. At the same time, FSH acts as a survival factor during PF transition to primary/secondary follicles in serum-free ovarian cortical tissue culture [11]. Studies in hamster using anti-FSH antibodies have shown that FSH action is also required for formation of primordial follicles [12]. They showed that a single dose of FSH specific polyclonal antibody during gestational period could significantly affect the number of primordial follicles after birth. Demeestere et al. [13] recently concluded that FSH possibly coordinates development of both germline and somatic compartments of the mouse follicle. FSH is also known to exert similar dual effect on both the germline and somatic cells in the testis. Rao et al. [14] reported that by blocking the action of FSH using phage-expressed extracellular domain of FSHR results in azoospermia after 100 days in non-human primates although FSHR are localized only on the surface of Sertoli cells. It remains rather unclear as to how FSH can exert action on both somatic and germline compartment of the ovarian follicles and in the testis, although FSHR are expressed only on the somatic cells namely granulosa cells of developing follicles and on Sertoli cells in the testis. Although indirect action on germ cells through paracrine-autocrine interactions is suggested, mechanism underlying FSH action on the mammalian gonads needs further investigation.

Several published reports using ovarian tissue or immortalized ovarian epithelial cell lines show that besides granulosa cells, FSH receptors (FSHR) are also localized on the normal ovary surface epithelium [15-17] and in ovarian tumor surface epithelium [9,18-26]. Oocytes have also been reported to express FSHR. FSHR mRNA was detected in the oocytes and cleavage stage mouse embryos [27]. FSHR is reported to be 20 fold higher in the human oocytes compared to granulosa cells [28]. Various techniques like *in situ* hybridization, RT-PCR, Q- PCR and immunolocalization studies have been used to demonstrate FSHR in the OSE of mice, rats, cows and humans [9,15,17,19]. Few studies have also shown that OSE undergoes rapid proliferation in response to PMSG [29-31], based on BrdU uptake or PCNA staining. All these studies suggest that FSH acting through FSHR localized in OSE may have an important role in ovarian biology. It is imperative to mention here that these published reports assume greater significance, since recently it has been reported by several groups [32-38] that the OSE also houses ovarian stem cells which may contribute to postnatal oogenesis. Hence, PMSG induced FSH action in adult mouse can serve as a good model to understand the probable mechanism of FSH action on stem cells as well as in new follicular development. Our group earlier reported that OSE in mice, rabbits, sheep, monkeys and women harbors two distinct stem cell populations that include the pluripotent very small embryonic-like stem cells (VSELs) and their immediate descendants ‘progenitors’ ovarian germ stem cells (OGSCs) [32,39]. We have further implicated these stem cells in postnatal oogenesis and proposed a working model for the same [39].

The concept of postnatal oogenesis in mammalian ovary is a highly debated area of research and several investigators have contributed both in favor [36,39-42] and against [43,44] in the recent past. Tilly’s and co-workers [36] were the initial group to generate evidence against the concept of fixed number of eggs at the time of birth and their recent paper [45] provides unequivocal evidence to support the presence of mitotically active germ cells that can generate oocytes both *in vitro* and *in vivo.* The existing controversy regarding existence of stem cells in adult mammalian ovary appears to be more of technical nature [46] or markers [47] used to study the stem cells rather than absence of stem cells itself. Recently, Gamwell and co-workers [48] recently reported a side population in mouse OSE that excludes Hoechst dye and express Sca-1 along with Nanog and Kit.

The present study was undertaken to study the effect of PMSG on mouse ovary with a focus on stem cells located in the OSE. Besides normal estrus cycle, ovaries were studied 2 and 7 days after PMSG treatment. Normally 2 days are required to stimulate maturation of follicles in a standard super-ovulation protocol using PMSG and hence 2 days was chosen to study the standard effect. A longer time period of 7 days was chosen to understand delayed effect and to get insights into the effect of FSH on ovarian stem cells. What is the effect of FSH when the ovary is super-ovulated during assisted reproduction, how does FSH treatment affect the OSE, does it have any effect on the stem cells and whether postnatal oogenesis and PF assembly is affected by FSH treatment are open questions that remain to be answered.

## Materials & methods

The study was approved by Institute Animal Ethics Committee and was carried out using in-house bred Swiss mice in the experimental animal facility at our Institute. Mice were housed in a temperature and humidity controlled room on a 12hr light/12hr darkness cycle with free access to food and water.

Different stages of estrus cycle were monitored by daily examination of vaginal smears of one month old female mice. Briefly, the vaginal swabs were prepared using sterile PBS dipped cotton swabs, fixed in 100% methanol, stained with Giemsa and observed under a 90i microscope (Nikon, Tokyo, Japan). Ovaries were collected during different stages of estrus cycle namely estrus, proestrus and diestrus (DE). Ovaries were also collected from mice treated with subcutaneous injection of 5IU PMSG (National Hormone & Peptide Program, Harbor-UCLA Medical Center, California, USA) after 2 days (2D) and 7 days (7D) of treatment. All PMSG injections were done in normal cycling mice at DE stage and mice at DE stage were used as controls for the experiments. The experimental design and number of animals used in the present study are shown in Table 1. The collected ovaries were fixed in 4% formalin for histology and immunolocalization studies and also in TRIZOL for RNA extraction to analyze various gene transcripts of interest by RT-PCR and Q-PCR. The ovaries were carefully dissected from all the adhering extraneous tissue under a stereomicroscope with the help of a micro scissor prior to freezing for RNA.

**Table 1:** Experimental design of the study

**Stage**	**Total number of animals**	**Number of ovaries**		
		**Total**	**Histology and IHC**^**§**^	**RNA isolation**^**#**^
Estrous	2	4	4	-
Pro-estrus	1	2	2	-
Di-estrus	10^*^	20	8	12
2D PMSG	12^*^	24	14	10
7D PMSG	12^*^	24	14	10

* Total number of animals used in 4 different experiments.

^§^ Whole ovary was sectioned and every third slide was used for H&E staining. The rest of the slides were used for IHC.

#Two ovaries from two animals were pooled to make one sample for RNA isolation. Only samples showing good A_260_/A_280_ ratio after RNA isolation were used for RT/Q-PCR study.

### Histological studies

Formalin fixed ovaries were processed and embedded in paraffin using standard protocols. 5μm thick sections of the embedded ovaries were prepared and stained with Hematoxylin and Eosin for studying the histo- architecture during different phases of estrus cycle and after treatment with PMSG. The representative areas were photographed using 90i microscope and data recorded.

### Immunohistochemistry (IHC)

Immunolocalization studies were carried out for various antigens listed in Table 2. Briefly, the paraffin sections were deparaffinized, hydrated and antigen retrieval was performed with boiling SSC buffer of pH 6 in microwave for 5 min (cytoplasmic antigens) or 15 min (nuclear antigens). After cooling, the slides were washed with Tris buffer saline (TBS) for 5 min and processed further for blocking with 10% NGS and 1% BSA in TBS. After removing excess blocking reagent, the slides were incubated with primary antibody diluted with 1% BSA in TBS for two hrs at room temperature. For negative control, the antibody was omitted and sections were incubated with 1% BSA in TBS. In case of FSHR detection alone, negative control included the pre-immune sera from rabbit in which the primary antibody was raised. The detection was done using anti mouse Vecta ABC kit (Vector Laboratories, USA) according to manufacturer’s instructions. Final color development was done using DAB (Biogenex, USA). After counterstaining with Haematoxylin, the slides were observed under 90i microscope. Representative areas were photographed.

**Table 2:** Details of antibodies used for immunolocalization studies

**Antibody**	**Marker**	**Source**	**Dilution**	**Reason to conduct IHC**	**Ref**
FSHR Polyclonal	FSHR: membrane bound receptor of FSH	Gift	1:100	To study cells responsive to PMSG	[49]
PCNA Monoclonal	PCNA: nuclear protein that functions during DNA replication	Sigma	1:5000	To study proliferating cells	[50]
OCT-4 Polyclonal	OCT-4 isoforms: OCT-4A is a nuclear transcription factor and OCT-4B is cytoplasmic	Abcam	1:100	To study pluripotent and progenitor stem cells	[32]
SCP3 Polyclonal	SCP3: a nuclear protein required for homologous recombination during meiosis I	Abcam	1:500	To study presence of cells undergoing meiosis	[36]
VASA Polyclonal	MVH: germ cell specific cytoplasmic protein expressed from primordial germ cell stage	R&D System	1:500	To study early germ cells and oocytes	[51,52]

### RNA isolation and cDNA synthesis

Total RNA was extracted by standard protocol using Trizol (Invitrogen, Carlsbad- CA, USA) and treated with DNase I (Amersham Biosciences, Piscataway, NJ) to remove any genomic DNA present. First-strand cDNA was synthesized using the iScript cDNA synthesis kit (Bio-Rad, USA) according to the manufacturer’s instructions. Briefly, 1μg of total RNA was incubated with 5x reaction mix and reverse transcriptase mix. The reaction was carried out in G-STORM thermocycler (Gene Technologies, Braintree, UK). The reaction mix was first incubated at 25°C for 5 min, then at 42°C for 30 min and finally at 85°C for 5 min.

Both RT-PCR and Q-PCR were employed to study gene transcripts expression for the following markers: *pluripotent stem cell* specific markers (**Oct-4A**: isoform A of octamer binding transcription factor 4 [53,54] and **Nanog:** homeobox transcription factor also expressed in primordial germ cells [55]); *early primordial germ cell markers* (**Oct-4**, represented here as total Oct-4 as it includes all isoforms of Oct-4 including isoform B expressed in cytoplasm of OGSCs [32]), **Stella**[56], **Fragilis**[57], **Mvh** mouse vasa homolog, plays a role in oogenesis, germ cell and follicle development); *oocyte specific markers* (**Nobox** newborn ovary homeobox-encoding gene is essential for folliculogenesis [58], **HoxA10** homoeobox gene restricted to primordial and early primary follicular oocytes [58]); *pre-meiotic marker* (**Stra8** stimulated by retinoic acid gene 8 essential for meiotic initiation [59]); *meiotic marker* (**Scp3** synaptonemal complex protein 3 and **Dmc1** required for, meiosis specific homologous recombination) and PMSG action mediator (**Fshr**). Each sample was obtained by pooling ovaries from two different animals.

### Reverse –transcription PCR

RT-PCR was carried out to study the expression of meiotic markers (Stra 8, Scp3 and Dmc1) using Dream Taq DNA polymerase (Fermentas Life Sciences; Vilnius, Lithuania). Briefly, the cDNA mix (2 μl) was amplified using 0.2mM of each primer (Table 3), 1.25 unit of DreamTaq DNA polymerase (Fermentas) in 1x Dream Taq buffer (Fermentas) and 0.2mM dNTPs in a G-STORM thermocycler. Amplification was carried out for 35 cycles, with each cycle consisting of denaturation at 94°C for 30 sec, annealing at the specified temperature (Table 3) for 20 sec, and extension at 72°C for 30 sec. The products were analyzed on 2% agarose gel stained with 0.5 μg/ml ethidium bromide (Bangalore Genei, Bangalore, India). The product size was approximated using a 100-bp DNA ladder (Bangalore Genei). The negative control did not include cDNA in the reaction mixture. Normal adult mouse testis was used as positive control.

**Table 3:** Primers used for RT- PCR and Q-PCR

**Gene**	**Primer**	**Annealing Temp (° C)**
**Fshr**	F: TGGAGGCGGCAAACCTCTGAAC	65
	R: TCTGGCTTTGGCGAGCAGGTC	
**Oct-4A**	F: CCATGTCCGCCCGCATACGA	61
	R: GGGCTTTCATGTCCTGGGACTCCT	
Total	F: CCTGGGCGTTCTCTTTGGAAAGGTG	68
Oct-4	R: GCCTGCACCAGGGTCTCCGA	
**Nanog**[34]	F: CAGGAGTTTGAGGGTAGCTC	61
	R: CGGTTCATCATGGTACAGTC	
Stella	F: ACGCTTTGGATGATACAGACGTCC	67
	R: GCGCTTTGAACTTCCCTCCGGA	
Fragilis	F: GGGGTGACTGAGCTGGGGGAA	65
	R: TGTCCCTAGACTTCACAGAGTAGGC	
Nobox	F: AGGGTGCTGAGAGGGTGGCAG	66
	R: GGCGATACTAGTGCCCCAGGAC	
HoxA10	F:CACTTGTCCGGCACCCCTTCG	65
	R: TTCGCCTTTGGAACTGCCTTGAC	
Mvh	F: AGTGGAAGTGGTCGAGGTGGT	61
	R: TGCCGGTGGTGCATCATGTCC	
Scp3	F: TGTTGCAGCAGTGGGAACTGGAT	68
	R: CCATCTCTTGCTGCTGAGTTTCCA	
Dmc-1	F: TGGGGAATTGGCTGAACGCC	68
	R: CAGGCATCTCGGGGCTGTCATAA	
Stra8	F: GTTTCCTGCGTGTTCCACAAG	61
	R: CACCCGAGGCTCAAGCTTC	
18s	F:GGAGAGGGAGCCTGAGAAAC	61
	R: CCTCCAATGGATCCTCGTTA	
Gapdh [34]	F: GTCCCGTAGACAAAATGGTGA	58
	R: TGCATTGCTGACAATCTTGAG	

### Quantitative PCR

Q-PCR was carried out to study the expression of various gene transcripts mentioned in Table 3. The expression levels of these gene transcripts in relation to housekeeping gene transcript 18s were estimated by CFX96 Real-time PCR system (Bio-Rad Laboratories, Hercules, CA) using SYBR Green chemistry (Bio-Rad).

The primers and their respective annealing temperatures used in the study are mentioned in Table 3. All the primers had efficiency close to 100% and all primers except Gapdh and Nanog are exon-exon spanning primers. The amplification conditions included initial denaturation at 94°C for 3 min followed by 40 cycles comprising of denaturation at 94°C for 30 secs, primer annealing for 20 secs and extension at 72°C for 30 secs. The final step included incubation at 94°C for 20 secs to remove any secondary structures followed by melt curve analysis. The fluorescence emitted at each cycle was collected during the extension step of each cycle. The homogeneity of the PCR amplicons was verified by running the products on 2% agarose gels and also by studying the melt curve. All PCR amplifications were carried out in duplicate. Mean Ct values generated in each experiment using the CFX Manager software (Bio-Rad) were used to calculate the mRNA expression levels. Since ΔCt is inversely proportional to relative mRNA expression levels, the levels were calculated manually by the ΔCt method.

## Results

### Histological studies

#### Ovarian surface epithelium (OSE) during different stages of estrus cycle

**Figure 1:**
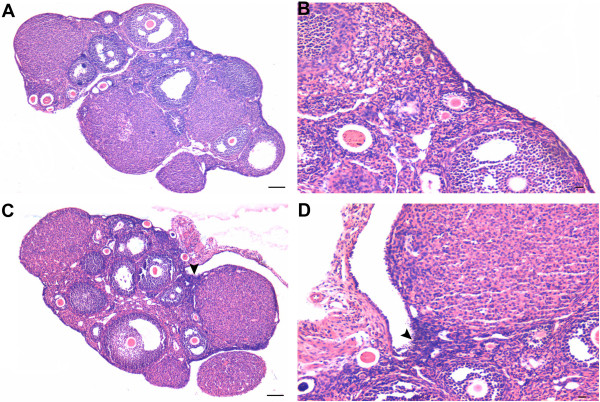
**Whole ovary histo-architecture during different stages of estrus cycle in one month old Swiss mice.** Follicles in different stages of development and corpus lutea can be observed. **A** &**B** During di-estrus stage, the ovarian surface is covered with cuboidal or flat epithelial cells. **C** &**D** During pro-estrus stage, a small area of the surface epithelial crypt reveals the presence of columnar epithelial cells (arrowhead). Bar: A&C-100μm, B&D-20μm.

**Figure 2:**
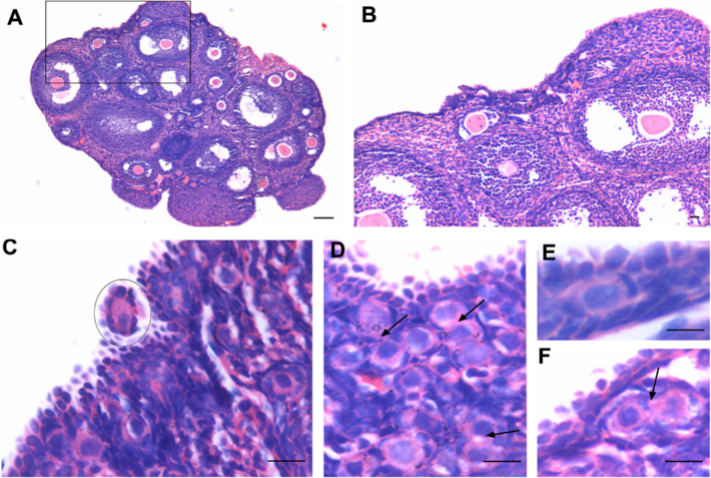
**Whole ovary histo-architecture during estrus stage of the cycle in one month old Swiss mice.A** Small area of OSE shows the presence of columnar epithelial cells giving highly folded multi-layered appearance (box). **B** Under higher magnification a cohort of primordial follicles (PF) are observed in the area where OSE exhibits proliferation. **C**-**F** The PF have cytoplasmic connections (arrow) between them. At places one can observe PF just below the OSE. PF assembly is evident in the OSE (circle). Bar: A-100μm, B-F-20μm.

Majority of epithelium covering the ovarian surface during different stages of the estrus cycle comprised of cuboidal or flat epithelial cells (Figure 1 &2). In comparison to diestrus stage (Figure 1 A & B) where OSE was cuboidal to flat, small regions of OSE appeared prominent and highly folded, gave multilayered appearance in proestrus and estrus stages (Figure 1C & D, Figure 2 respectively). Cohorts of PFs were invariably visualized under this region of OSE, especially during the estrus stage. At places, relatively large cells with pale cytoplasm, surrounded by a single layer of somatic granulosa-like cells were observed in the OSE (Figure 2 C-F). The cohorts of PFs appeared to have cytoplasmic continuity at places (Figure 2 D & F). It appeared as if PF undergo follicular assembly in the OSE and then get pinched off from the OSE and shift to the cortex region just below the OSE always in a cohort.

#### Ovarian surface epithelium after two (2D) and seven (7D) days post PMSG treatment

**Figure 3:**
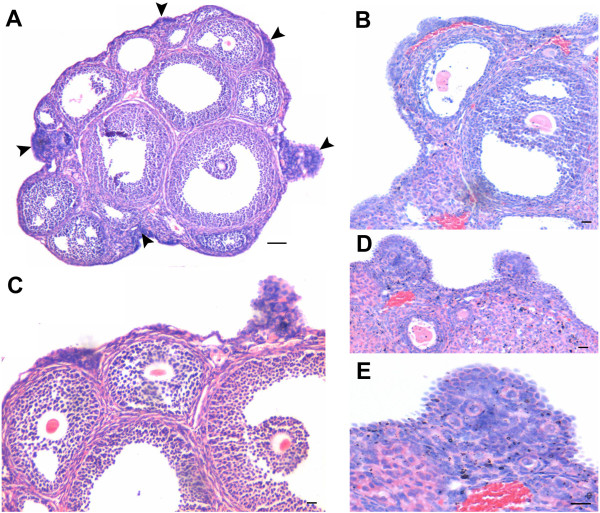
**Ovary histo-architecture after two days of PMSG treatment.** As compared to estrus stage, two days of PMSG treatment results in increased proliferation of OSE at several places (arrowhead). A large number of mature, unruptured follicles are observed and corpus lutea are absent. There is slight increase in vascularization just below the OSE. The small protuberances on the ovarian surface are marked by epithelial cells present in multi-layer and have several PF within them. Bar: A-100μm, B-E-20μm.

**Figure 4:**
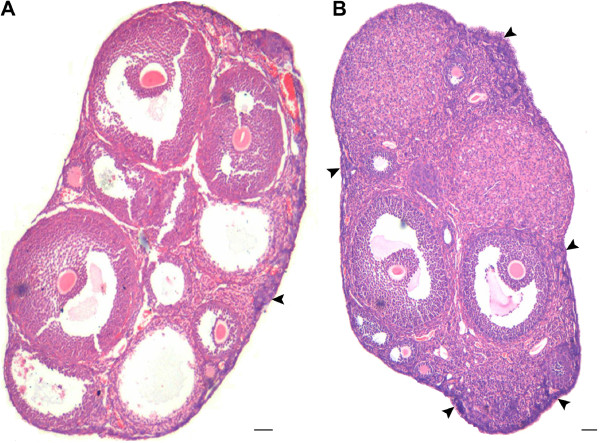
**A comparative histo-architecture of mouse ovary during normal estrus stage and 7 days after PMSG treatment.** As evident, focal areas where OSE gives multilayer appearance and has associated cohort of PF are markedly increased after PMSG treatment **B** (arrow head) as compared to control **A** Bar: 100μm.

**Figure 5:**
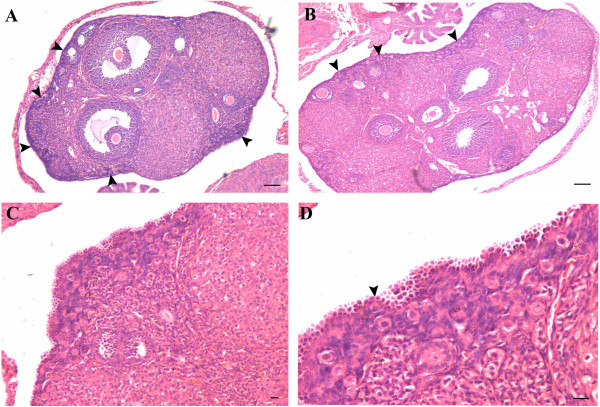
**Ovarian histo-architecture after 7 days of PMSG treatment.A** &**B** Whole ovary sections showing several focal areas with multilayered OSE and associated cohorts of PF. **C** &**D** Higher magnification of the region with multilayer OSE and PF. PF assembly in the OSE is evident (arrowhead). Bar: A&B-100μm, C&D-20μm.

**Figure 6:**
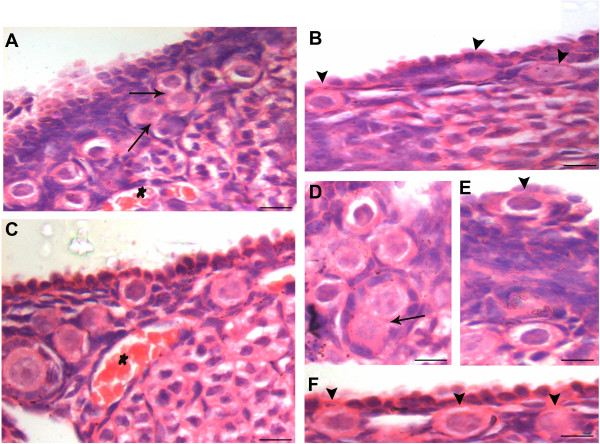
**Higher magnification of D7 PMSG treated H & E stained ovarian sections.** The epithelial cells appear proliferative and multilayered. A cohort of PF is invariably observed below such regions of OSE. Note increased vascularity in this region (star) and cytoplasmic connection between the oocytes of two adjacent PF (arrows). The PF assembly occurs in the OSE (arrowhead). The oocytes are large in size with abundant pale cytoplasm whereas granulosa cells have more fibroblast-like appearance and are observed surrounding the oocytes. Bar: 20μm.

PMSG had a profound effect on OSE in addition to its known effect on growing cohort of follicles. 2D after PMSG treatment, OSE exhibited extensive proliferation in agreement with earlier reports [29-31]. We also observed OSE having multilayered appearance along with the presence of small outgrowths, which enclosed many PFs (Figure 3). Increased vascularity near the OSE was also observed. The ovaries from 7D PMSG treated mice showed striking difference when compared at lower magnification to ovary from control (Figure 4 &5). As evident, in control ovarian section during the estrus phase, only a single small region of OSE showed proliferation, had multilayer appearance and a cohort of PF was observed (Figure 4A). In contrast, the OSE after 7D of PMSG revealed the presence of 5–8 regions where the OSE was of hyper proliferative nature accompanied with the presence of several cohorts of PF (Figure 4B, Figure 5A & B). However, in comparison to 2D, the protuberances in OSE were reduced on 7D. At places, PF assembly was evident in OSE (Figure 5 &6) wherein a large germ cell with abundant pale cytoplasm was observed in the OSE. Nests of follicles were also observed with prominent ooplasm continuity. It appeared that after PF assembly, these PF were pinched off and shifted to the ovarian cortex just below the OSE, similar to control ovaries in the estrus phase (Figure 2). However, PMSG exposure accelerated the frequency of the whole process of oogenesis and PF assembly.

### Immunolocalization studies

#### FSH receptors

**Figure 7:**
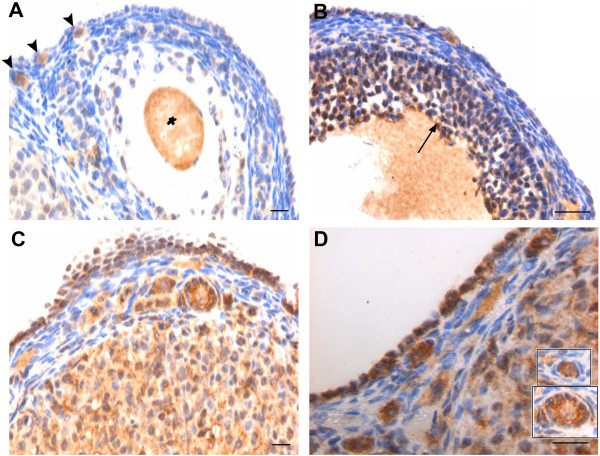
**Follicle stimulating hormone receptor (FSHR) immunolocalization in estrus stage and after 7D of PMSG treatment.A** &**B** In control, minimal staining is observed in the OSE cells. However, distinct immunolocalization is observed in the oocyte of the PF (black arrow), whereas the surrounding granulosa cells are negative. The granulosa cells in large follicles show positive staining (blue arrow). Positive staining is also observed in the oocyte in large follicle (asterisk). **C** &**D** Increased immunostaining pattern is observed after PMSG treatment in the multilayered OSE, oocytes of primordial and developing primary to secondary follicles. Inserts show follicles in various developmental stages with granulosa cells negative for FSHR. Note change in shape of the granulosa cells from flat to cuboidal as the PF transits into a primary follicle. Bar=20μm.

FSHR immunolocalization was relatively less in control ovarian sections (Figure 7A & B). Minimal staining was observed in OSE, oocytes and corpus luteal cells whereas dark and prominent staining was observed in the granulosa cells of mature follicles (Figure 7B). The oocyte also showed positive immunostaining for FSHR (Figure 7A & B). PMSG treatment had a profound effect on FSHR expression pattern in the ovarian sections (Figure 7C-D). An increased staining was observed in granulosa cells, OSE cells (Figure 6C) and oocytes of primordial, primary and secondary follicles. The granulosa cells in primordial follicle were distinctly negative for FSHR.

#### PCNA

**Figure 8:**
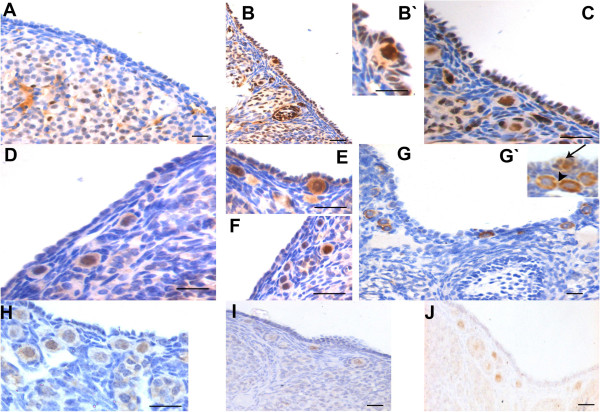
**Immuno-staining on control and PMSG treated ovarian sections.A**-**C**: Immunostaining with anti-PCNA antibody. Note minimal PCNA staining in control OSE. Increased staining is observed after PMSG treatment in both the OSE cells and the oocytes of PF (**B**-&**C**). Inset **B**’ is magnified image of the PF located in the OSE. At places, granulosa cells were also positive for PCNA (arrowhead). **D**-**F**: Immunostaining with anti-OCT-4 antibody in control (**D**) and 7D PMSG treated (**E**&**F**) ovarian sections. OCT-4 is localized in the ooplasm in control, while in 7D PMSD treated, positive staining was observed in ooplasm as well as in the nucleus of few oocytes (arrowhead) in PFs. **G**: Immunostaining with anti-human VASA antibody that cross-reacts with mouse MVH. MVH is distinctly localized in the ooplasm of PF and at places was also observed in a ‘germ cell nest’ (**G**’, arrow). At places some oocytes of primordial follicles in cohorts appeared connected without intervening granulosa cell (arrowhead) **H**-**J**: SCP-3 is localized in PF oocytes present in close vicinity of multilayer OSE. Bar: 20μm.

As compared to control (Figure 8A), PMSG treated OSE (Figure 8B & C) showed increased PCNA staining. The germ cell nuclei of PFs in the OSE also showed prominent staining suggesting the PFs were newly assembled (Figure 8B’). PCNA labeling was also observed in the granulosa cells of developing follicles. As expected, the oocytes of bigger follicles were negative for PCNA (data not shown).

#### OCT-4

In control samples, faint OCT-4 staining was observed in the ooplasm of primordial follicles (Figure 8D). In 7D PMSG treated ovarian sections, OCT-4 staining was more intense and the PFs lying below the OSE showed both nuclear (arrowhead) and cytoplasmic staining for OCT-4. However, the surrounding granulosa cells were distinctly negative for OCT-4 (Figure 8 E & F) in both control and after PMSG treatment.

#### MVH

MVH (a proliferating germ cell and oocyte marker) was localized in the ooplasm of primordial to secondary follicular oocytes (Figure 8G). Some of the primordial follicles in cohorts still appeared cytoplasmically connected without any intervening granulosa cell (Figure 8G’, arrowhead) and exhibited positive MVH staining. Also at places cluster of germ cells in the OSE also stained positive for MVH (Figure 8G’, arrow), probably representing mitotically dividing pre-meiotic germ cell nests, which are well documented in perinatal mouse ovary [60].

#### SCP3

SCP3, a meiotic marker was distinctly immuno-localized in the nuclei of some oocytes along OSE after PMSG treatment. The staining of SCP3 was evenly localized throughout the nuclei in 7D post PMSG (Figure 8 H-J) suggesting oocytes in meiosis prophase I. Nucleolar staining was occasionally observed in primordial follicles of control as well as in 2D post treatment (data not shown).

### Gene transcript analysis

#### RT-PCR

**Figure 9:**
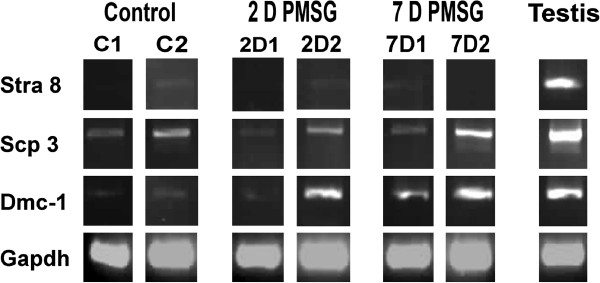
**RT-PCR results for pre-meiotic (Stra8) and meiotic (Scp3, Dmc-1) markers in control and PMSG treated ovaries along with testis as positive control.** Note Stra8 is observed only in control sample C2, 2D PMSG sample 2D2 and 7D PMSG sample 7D2 at very low levels compared to testicular sample. Meiotic marker Scp3 and Dmc1 are upregulated in PMSG treated samples. Each sample was obtained by pooling ovaries from two animals.

Figure 9 shows RT-PCR results for pre-meiotic and meiotic markers using normal adult testicular tissue as positive control. Only few of the samples namely control C2, 2D2 and 7D1 of PMSG treated samples showed faint positivity for Stra8 when compared to brightly positive band from control testicular samples. This may be because very few cells in whole ovary undergo meiosis and normally STRA8 positive cells are rarely detected in young adults [61]. Niikura et al. [61] suggested that this may reflect a quick transition of pre-meiotic germ cells, once committed by inducing *Stra8* expression, into oocytes during young adulthood. On the other hand, meiotic markers Scp3 and Dmc1 were detected in all the samples studied, albeit at very low levels in control samples. This suggests meiosis occurrence at extremely low level in normal adult ovary, while it is upregulated during PMSG treatment. To further confirm this, Q-PCR analysis of Scp3 was performed.

#### Q-PCR analysis of various transcripts

**Figure 10:**
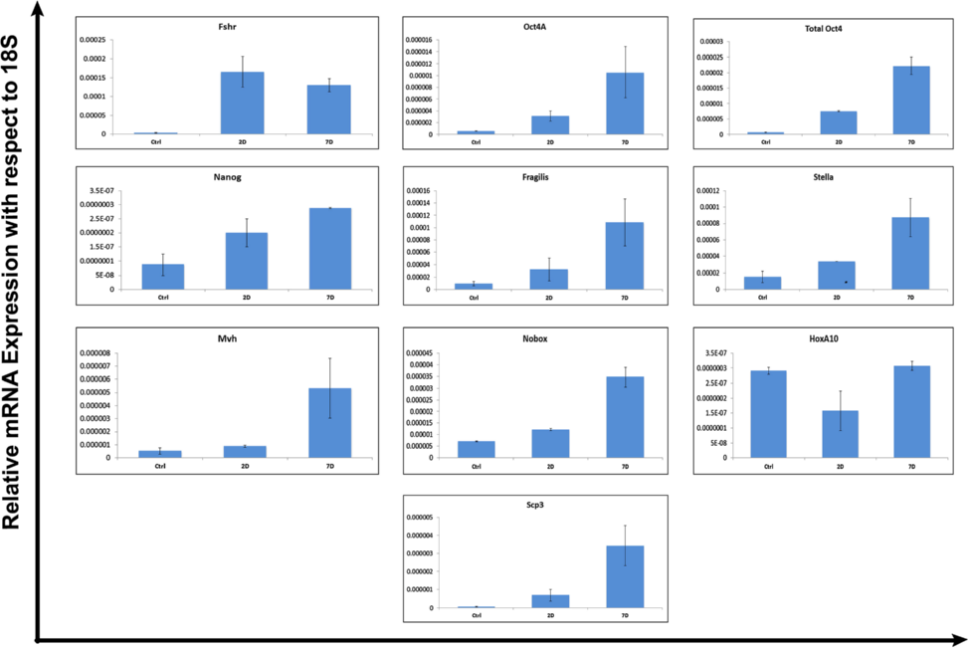
**Q-PCR analysis of various gene transcripts in ovaries from control, 2D and 7D after PMSG treated mice.** Note an increase in Fshr mRNA expression after PMSG treatment on D2, which slightly reduced by 7D of PMSG treatment. All other markers specific for pluripotent stem cells, germ cells, oocytes and meiosis showed an increase in mRNA expression after PMSG treatment to varying degree except for HoxA10. Increase in pluripotent (Oct-4A and Nanog) and meiotic marker (Scp3) after PMSG treatment suggest that the VSELs get stimulated by PMSG treatment, undergo further proliferation, differentiation and meiosis leading to neo-oogenesis and PF assembly. Each sample was obtained by pooling ovaries from two animals.

The Q-PCR data obtained by analysis of control and PMSG treated ovaries is presented (Figure 10). As expected, a significant increase in Fshr mRNA expression was noted on 2D after PMSG treatment compared to control sample and showed slight reduction by 7D. PMSG treatment also led to increased stem cell activity as evident by increase in expression of stem cell and primordial germ cell markers including Oct-4A, Nanog, total Oct-4, Fragilis and Stella on D2 after PMSG and were further increased or sustained by D7. As mentioned earlier, we have previously reported that the pluripotent VSELs have nuclear OCT-4 (specific transcript is Oct-4A) and once they initiate differentiation, OCT-4 shifts to cytoplasm in OGSCs [32]. Results of the present study indicate that the increase in total Oct-4 post PMSG treatment was more than the increase in Oct-4A (Figure 10), suggesting that OGSCs are also proliferating post-PMSG treatment. Also increase in expression was observed in germ cell marker (Mvh) and interestingly significant increase in Nobox was maximally observed on 7D. HoxA10 was decreased on 2D post treatment probably because of maturation of pre-existing primordial and primary follicles. Interestingly, the HoxA10 levels increased on 7D and reached levels comparable to control suggesting formation of new primordial follicles post PMSG treatment. RT-PCR and Q-PCR analysis for the meiotic marker Scp3 also showed a significant increase 7D after PMSG treatment suggesting increase in meiosis post treatment.

## Discussion

Present study demonstrates that pluripotent stem cells VSELs and progenitors OGSCs reported earlier by our group in adult mice, rabbit, sheep, monkey and human ovary [32,39] are possibly regulated by FSH and are responsible for postnatal oogenesis and follicular assembly during adult reproductive life. Normal adult mice ovaries were studied during different stages of estrus cycle and after 2 and 7 days of PMSG treatment by histology, immunolocalization (FSHR, PCNA, OCT-4, SCP3 and MVH), and RT-PCR/ Q-PCR for various gene transcripts including pluripotent (Oct-4A, Nanog); early germ cell (total Oct-4, Stella, Fragilis, Mvh), oocyte-specific (Nobox & HoxA10); pre-meiosis (Stra8) and meiosis (Scp3, Dmc1) specific markers. The process of ovarian stem cell differentiation and meiosis giving rise to new oocytes, which get enclosed by the granulosa cells to assemble as PFs in the OSE appears to be a normal feature of adult ovary (Figure 1,2). The granulosa cells arise by epithelial-mesenchymal transition of OSE as suggested earlier by us based on *in vitro* evidence [32]. Present study provides *in vivo* evidence in favor of our earlier observations that OSE cells may be involved in the formation of granulosa cells (Figure 6B,C, E & F). PMSG treatment further augments the process of postnatal oogenesis and PF assembly resulting in increased PFs by D7 (Figure 3, 4, 5, 6).

PMSG is known to exert its actions on mammalian ovary through FSH-FSHR interaction. In the present study, besides granulosa cells of bigger follicles, FSHR was detected in both OSE and oocytes of PF in agreement with earlier reports [15-17,19,27,28] and an up-regulation was observed after PMSG treatment at both protein by immunohistochemistry (Figure 7) and transcript (Figure 10) level. Presence of FSHR in locations like oocytes and OSE suggests that the various diverse functions of FSH are probably not exerted only through the classical FSHR present on the granulosa cells of growing follicles. Sairam’s group has earlier reported that FSH may exert diverse actions through alternatively spliced FSHR isoforms based on their studies from sheep and mice ovaries [62-64].

In the current study, upregulation of FSHR after PMSG treatment correlated well to the proliferation observed in OSE. OSE proliferation and multilayer appearance which were normally observed in control ovaries at few focal areas were increased after PMSG treatment (Figure 3 &4). Ovarian sections on 2D of PMSG treatment showed the presence hyperplastic epithelial cells and small protuberances bulging from the ovarian surface (Figure 3) which subside by 7D (Figure 4, 5, 6). PF cohorts normally observed with hyper-proliferative OSE regions in control ovaries, increased significantly after 7D of PMSG treatment in the ovarian cortex (Figure 4 &5). This correlated well with increased mRNA expression for HoxA10- a primordial follicular oocyte marker [58] and Nobox that is expressed abundantly in primordial and primary follicular oocytes [58] post PMSG treatment. Occurrence of meiosis was supported by expression of pre-meiotic (Stra8) and meiotic (Scp3 and Dmc 1) markers. All the three markers were detected in control as well as all PMSG treated ovarian samples. A distinct up regulation of Scp3 was noted after PMSG treatment, both at the protein (Figure 8 H-J) and mRNA transcript level (Figure 9 &10). As reported earlier [65], SCP3 staining was observed throughout the nuclei suggesting that the oocytes are in prophase I of meiosis and was gradually lost in more developed oocytes. The PF at birth are generally arrested in diplotene stage of prophase 1 of meiosis. SCP3 expression is restricted to earlier stages i.e. zygotene and pachytene stage. Thus expression of Scp3 and pre-meiotic marker Stra8 in adult ovary are considered direct evidence in support of postnatal oogenesis and have been reported earlier by other groups also [36,66].

It is intriguing to point out that OSE which expresses FSHR also lodges VSELs as observed in rabbit, sheep, monkey, human [32] and also mouse ovaries [39]. The VSELs with nuclear OCT-4 differentiate into ‘progenitors’ ovarian germ stem cells (OGSCs) with cytoplasmic OCT-4 and undergo spontaneous differentiation into oocyte-like structures *in vitro*[32,37]. Possibly, in the present study, the pluripotent VSELs were activated by PMSG treatment (increased expression of Oct-4A, Nanog Figure 8 &10), undergo proliferation (increased PCNA staining and Oct-4A expression, Figure 8) and differentiation (increased expression of stella, fragilis, total Oct-4, Vasa and MVH Figure 8 &10) resulting in meiosis (Stra-8, Scp3 and Dmc1 Figure 8, 9, 10) and formation of oocyte nests with prominent intercellular cytoplasmic bridges that assemble to form PF in the OSE (Figure 2 &6). These nests later shift to the cortex and are visualized as small cohorts on 7D after PMSG treatment. This data demonstrates the mechanism of oogenesis occurring *in situ* in adult ovary for the first time in OSE similar to well-studied spermatogenesis in the testicular seminiferous tubules where the VSELs are lodged at the basement membrane [67,68]. The process is regulated by FSH and is in contradiction to the existing paradigm that initial PF growth is gonadotropin independent [69,70]. The results of the present study support the model for postnatal oogenesis proposed by us earlier [39].

Messinis et al. [70] have reviewed a huge body of literature and summarized the current understanding of the role played by FSH during folliculogenesis. According to existing understanding, the ‘intercycle peak’ of FSH is crucial and responsible for ‘cyclic recruitment’ of follicles. Ovary experiences waves of folliculogenesis and a cohort of follicles start growing in each cycle, however, only one becomes dominant and others undergo atresia [69]. On treating with gonadotropins, FSH window gets widened and multiple follicles get selected and start to grow. Whether more follicles get recruited due to the treatment or FSH prevents atresia in the already recruited cohort of PF is still controversial. Results of the present study add another dimension to this by demonstrating that FSH also exerts action on the OSE and induces neo-oogenesis and PF assembly from the stem cells residing in the OSE.

The prevailing notion of extrusion of atretic oocytes from the ovary surface [71-73] or that the PF may get pushed to the ovarian surface because of the growing follicles in response to the PMSG treatment (and thus increased number of PF are visualized after treatment in the present study) may not be valid since this reasoning cannot account for distinct increased pattern of pluripotent and meiotic markers in the developing follicles (Figure 7, 8, 9) observed in the present study. Also the earlier published reports of BrdU incorporation in OSE [34,36] may not be due to mtDNA synthesis – rather it may be due to stem cell activity that gets augmented by PMSG.

The extensive germ cell nest formation, initiation of meiosis, PF assembly etc. are well documented and studied during fetal ovarian development in the existing literature [60]. It is interesting to point out that the stage of fetal development when PF assembly occurs is actually associated with several folds higher levels of FSH in females [2-6,74]. Thus FSH is directly related to PF assembly and regular intercycle peak of FSH may be stimulating the VSELs to result in differentiation and PF assembly. Lei et al. [74] reported that the high levels of circulatory FSH in perinatal period and increased FSHR mRNA in mouse ovaries facilitate germline nest breakdown and PF assembly.

## Conclusions

We propose that PMSG treatment through induction of FSH-FSHR action possibly stimulates the pluripotent VSELs residing in the OSE leading to proliferation and differentiation of OGSCs to oocytes and primordial follicle assembly. These results are in contradiction to the existing paradigm that ovaries have fixed number of follicles in adulthood and that early PF growth is independent of FSH.

The results of the present study are in direct agreement with postnatal oogenesis proposed by Tilly’s group in 2004 [36]. We propose that intercycle FSH peak is probably associated with cyclic recruitment of VSELs in the OSE throughout reproductive life and results in neo-oogenesis and PF assembly. Several fold elevated FSH levels in female fetus during peri-natal period [2-6] are possibly responsible for increased PF assembly well documented during fetal development. Moreover, since VSELs exist in various body organs, it would be interesting to study whether only ovarian VSELs are selectively stimulated by FSH or even VSELs in testis [67,68] or bone marrow [75] etc. are also regulated by FSH. It is indeed intriguing that despite exploiting the ovaries for so many years as a source of eggs, present study has provided newer information towards the mechanism of action of FSH on the ovaries. Elevated FSHR3 (an isoform of FSHR) in OSE has been implicated in ovarian cancers [25]. It is highly likely that the VSELs residing in the OSE may also be implicated in the ovarian cancers as has been proposed earlier [76,77].

## Abbreviations

2D PMSG: 2 Days post PMSG treatment; 7D PMSG: 7 Days post PMSG treatment; DE: Diestrus; FSH: Follicle stimulating hormone; FSHR: Follicle stimulating hormone receptor; OGSCs: Ovarian germ stem cells; OSE: Ovarian surface epithelium; PF: Primordial follicle; PMSG: Pregnant mare serum gonadotropin; Q-PCR: Quantitative polymerase chain reaction; RT-PCR: Reverse transcription polymerase chain reaction; VSELs: Very small embryonic like stem cell.

## Competing interests

Authors declare no competing interests.

## Authors’ contributions

DB was responsible for conceptualizing the study, data interpretation and manuscript preparation. KS was responsible for RT PCR and Q PCR analysis, data compilation and manuscript preparation. PG was responsible for immunolocalization studies and data compilation. HM was responsible for histology studies. All read and approve the final manuscript.
